# National COVID-19 Vaccine Program and Parent’s Perception to Vaccinate Their Children: A Cross-Sectional Study

**DOI:** 10.3390/vaccines10020168

**Published:** 2022-01-22

**Authors:** Thamir Al-khlaiwi, Sultan Ayoub Meo, Hamad Abdulaziz Almousa, Abdulrahman Ahmed Almebki, Mansour Khalid Albawardy, Hassan Haider Alshurafa, Meshal Abdulaziz Althunayan, Mohammed Sulaiman Alsayyari

**Affiliations:** 1 Department of Physiology, College of Medicine, King Saud University, Riyadh 11461, Saudi Arabia; talkhlaiwi@ksu.edu.sa; 2 College of Medicine, King Saud University, Riyadh 11461, Saudi Arabia; Hamad@almousa.com (H.A.A.); aamobki@gmail.com (A.A.A.); M.K.albawardy@gmail.com (M.K.A.); Hassanshurafa35@gmail.com (H.H.A.); Mishalthu@gmail.com (M.A.A.); sayyari34@gmail.com (M.S.A.)

**Keywords:** vaccination of children, parent willingness, COVID-19, safety

## Abstract

Vaccinating children against COVID-19 is an essential public health strategy in order to reach herd immunity and prevent illness among children and adults. Parents are facing tremendous stress in relation to the COVID-19 pandemic and the effectiveness of the COVID-19 vaccination program for children. In this study, we aimed to investigate parents’ perceptions and acceptance of the COVID-19 vaccine for their children in Saudi Arabia. A well-designed, pre-validated, Google questionnaire was distributed to parents through social media websites. The selection of the participants was based on the simple random sample technique. The study sample size was 1304 participants, with 342 males (26.2%), and 962 females (73.8%). The personal information, perception about COVID-19, and their children’s vaccination status were obtained. Among the participants, 602 (46.1%) were willing to get the COVID-19 vaccination for their children, whereas 382 (29.3%) were hesitant to inoculate their children for COVID-19 and 320 (24.4%) were unsure. Age (*p* = 0.004), gender (*p* = 0.001), occupation (0.004), income (*p* = 0.030), and vaccination status (*p* = 0.001) had an influence on the parents’ acceptance of COVID-19 vaccination of their children. On the other hand, education level, number of children, and having been previously infected with COVID-19 had no statistically significant effect on the parent acceptance. The correlation of parents’ knowledge about COVID-19 and their agreement to the vaccination of their children was statistically significant, along with gender (males were more knowledgeable, with *p* < 0.001), occupation, income (higher income showed a statistical difference, with *p* < 0.001), and vaccination status (*p* < 0.001). There was a decrease in parents’ acceptance toward the COVID-19 children vaccine in Saudi Arabia, which requires more attention and focus from health providers to eliminate fear and anxiety among the parents through additional educational programs and events to decrease the resistance toward the vaccination of children. More emphasis is required to increase the awareness of parents and convey the importance of the vaccine for children. In addition, more studies are needed to ensure the vaccine’s safety.

## 1. Introduction

Nobody can deny the detrimental effects of the COVID-19 pandemic on health, social life, and the economy [1,2,3], and part of this effect takes place on children [4]. Studies have shown that most children have an asymptomatic infection or mild-to-moderately severe manifestations (5% of COVID-19 incidence) with an infrequent incidence of death [5,6,7,8,9,10]. However recent studies revealed that 11.5% of COVID-19 cases in the United States were children, and some developed multisystem inflammatory syndrome (MIS) [11]. It is also a fact that children can spread the disease [12]. All over the world, parents are directly responsible as decision-makers in relation to their children’s health issues. Without controlling the spread of the COVID-19 pandemic throughout all ages in society, the hope of ending this pandemic would be almost impossible [13,14,15,16].

During the COVID-19 pandemic, parents have significant concerns about the COVID-19 vaccine for their children, since some countries have approved the COVID-19 vaccine for those below 18 years. There is a great conflict between the government’s decisions regarding ending this pandemic and the fear among parents towards providing the vaccine to their children. In Saudi Arabia, health officials plan to offer COVID-19 vaccines for children to end the pandemic. Studies have been conducted to evaluate the views of parents in several countries; 65% of caregivers in the United States of America were willing to provide the COVID-19 vaccine to their children against COVID-19 [17]. Another study conducted in the United Kingdom revealed that 48.2% of parents agreed to provide COVID-19 vaccine to children below 18 years, and 40.9% were unsure but tended toward acceptance [18]. In China, the prevalence of parents’ acceptance of the COVID-19 vaccination for their children was 72.6% [19], and 80% of participants in New Zealand agreed on COVID-19 vaccination of children [20]. A cross-sectional study conducted in Turkey showed that 36.3% of parents were willing to give their children the COVID-19 vaccine [21], whereas another study in Turkey showed that 38.4% agreed to vaccinate their children [22].

In this study, we aimed to evaluate parents’ perceptions of COVID-19 vaccination of children in Saudi Arabia. Parents and children are facing tremendous stress during the pandemic and the effects of the media, which has impacts on mental and emotional states [23,24]. Therefore, it is of great value to improve our understanding of parents’ opinions on the COVID-19 vaccination of children. In addition, it is also essential to understand the reasons that lead to certain decisions taken by health officials to minimize any misconceptions about the vaccination program, which may provide more support towards making the right choices in combatting the COVID-19 pandemic.

## 2. Subjects and Methods

In this observational, cross-sectional questionnaire-based study, the sample was drawn from Saudi Arabian adult residents. The study relied on the random distribution of the survey among the study population. The sample size was estimated following the assumption that 75% of the parents may have a positive attitude [25], a confidence interval of 95%, and a margin error of ±5%. The sample size was calculated to be 1200 participants, which was adjusted up to 1300 to ensure the required number of responses, considering a 15–20% non-response rate. However, in this study, a sufficient sample size was included to provide the appropriate representation of the participants. We distributed 1754 questionnaires, and 1304 participants responded, and these were finally used for analysis.

The predesigned Google proforma was sent to each parent, including their personal information, opinions regarding the COVID-19 pandemic, and the COVID-19 vaccine for children. Ten surveys were distributed initially to participants to check for any technical issues and ensure the clarity of the questions. The 10 participants involved in the preliminary survey were not included in the actual study data analysis. After reviewing the reliability of the survey, the questionnaires were distributed through social media sites, family groups, and personal efforts.

The questionnaire included sociodemographic information sections, such as age, gender, education level, occupation, income, number of children, vaccination status, and whether the respondent has been infected with COVID-19. One question was included to measure parents’ acceptance of the COVID-19 vaccination of their children. A 3-point Likert scale was used (agree, not sure, disagree). In addition, we added questions that measured the respondents’ knowledge regarding COVID-19. The last part of the questionnaire regarded the reasons that directed parents toward the acceptance or rejection of the COVID-19 vaccination of their children.

The participants were informed that their participation was voluntary and anonymous. In addition, they were informed that by filling out the questionnaire, they were providing informed consent to participate in the study. All questions were mandatory to complete the questionnaire, and there were no missing data. The inclusion criteria were having at least one child aged 5–12 years and living in Saudi Arabia. The exclusion criteria were not having a child aged 5–12 years or not living in Saudi Arabia.

### Data Analysis and Ethical Approval

The data were analyzed using SPSS 25 version statistical software. The correlation was assessed between the variables and we quantified the degree to which these variables were related. Descriptive statistics (frequencies, percentages, means, and standard deviations) described the categorical and quantitative variables. Pearson’s Chi-squared test was used to assess the measure of association between categorical and outcome variables. A *p*-value of ≤0.05 and 95% confidence intervals were used to report the statistical significance. The Institutional Review Board, College of Medicine Research Centre, King Saud University, Riyadh, Saudi Arabia approved this study (Ref-E-21-6228).

## 3. Results

In this study, 1304 validated questionnaires were retrieved for analysis; 1052 (80.7%) of the participants were from Riyadh. The sociodemographic data are presented in Table 1, showing that there were 542 participants aged from 40 to 49 years, representing 41.6% of the sample. Regarding gender, 962 were female, representing 73.8% of the sample, and 882 (67.6%) had a bachelor’s degree. Nine hundred sixty-five (74%) of the participants had one or two children. Regarding occupation, 485 (37.2%) of the respondents were working in the government sector; 418 (32.1%) reported being unemployed. In regard to income, 448 (34.4%) reported earning between 15,000–24,999 Saudi Riyal (SR), and 393 (>30%) reported earning more than 25,000 Saudi Riyals per month. Out of one thousand three hundred and four (1304) participants, the majority (86%) of participants had been vaccinated with two doses, representing one thousand one hundred and twenty-one (1121) of the participants. However, 84 participants (6.4%) were immunized with one dose, and 32 participants (2.5%) were non-immunized with the vaccine. Additionally, sixty-four of the participants (4.9%) had been infected with COVID-19, and seven hundred forty-six (746) did not have any family members infected by the virus (57.2%).

As shown in Table 2, 602 (46.1%) of participants agreed to give the COVID-19 vaccine to their children, whereas 382 (29.3%) disagreed with providing the vaccine to their children, and 320 (24.4%) were not sure.

Table 2 shows a positive statistically significant relationship between age and willingness to vaccine their children (≥40 years, 422 participants, representing 70% with a *p*-value of 0.004). Furthermore, gender also showed a positive statistically significant association, with a *p*-value < 0.001, with 404 (67.1%) females agreeing to vaccinate their children, compared to 198 (32.9%) males. Moreover, occupation revealed a significant positive association, with a *p*-value of 0.004, as well as income, with *p* = 0.03. In addition, the vaccination status of the participants appeared to have a positive association, with a *p*-value less than <0.001. However, education level did not show a significant relationship (*p* = 0.250), nor did the number of children (*p* = 0.708).

Table 3 demonstrates the parents’ knowledge about the COVID-19 vaccine; information was recorded through the questions in the survey. The participants were asked about COVID-19, its impact on society, children, disease carriers, and the safety of COVID-19 vaccines for children. Knowledge about the vaccine showed a positively significant association with gender (male (79.5 ± 0.218), *p <* 0.001 compared with female (74.5 ± 0.213)); occupation (student (78.8 ± 0.244), private sector (78.6 ± 0.193), and governmental sector (76.9 ± 0.219) with *p =* 0.004); income (higher income showed a positive statistical difference—25,000–50,000 (80.0 ± 0.198) compared to more than 50,000 (79.9 ± 0.205) with *p <* 0.001), and vaccination status (vaccinated with two doses (77.7 ± 0.203) *p <* 0.001).

**Figure 1:**
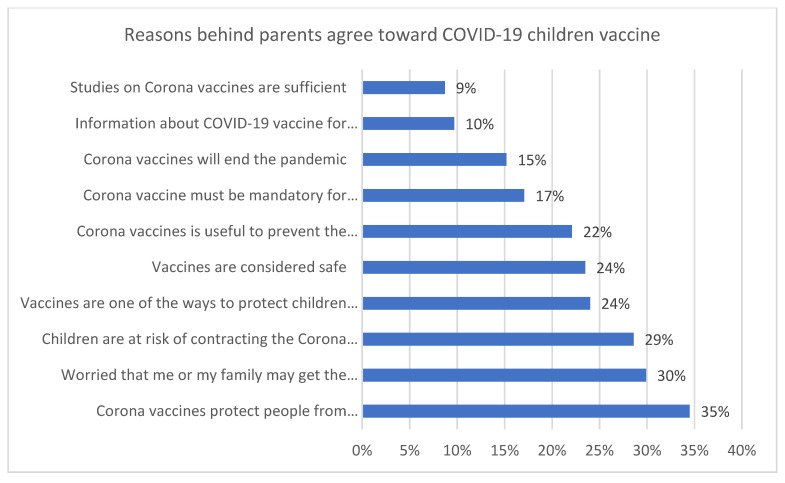
Reasons for parents’ agreement to vaccinate their children for COVID-19.

**Figure 2:**
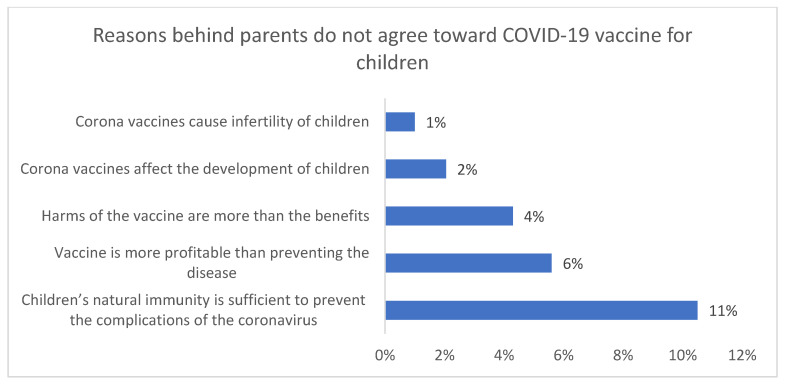
Reasons for parents’ disagreement to vaccinate their children for COVID-19.

Figure 1 shows that the most important reasons that directed parents toward the acceptance of the vaccine were that the vaccine could protect against the disease (n = 449, 34.5%), worry about oneself or one’s family member being infected (n = 378, 29.9%), and anxiety about one’s children being affected (n = 313, 24%). It also shows that 10% (n = 126) of participants thought the information regarding the COVID-19 vaccine for children was reliable. Figure 2 shows that an important reason behind refusing to vaccinate one’s children is the belief that children have natural immunity (n = 137, 11%). It also shows that the second reason that prevented parents from vaccinating their children was the believe that the goal of the vaccine was for profit, more than ending the pandemic (n = 73, 6%).

## 4. Discussion

The fear among people towards vaccines is one of the obstacles of this pandemic [25]. Since the recent approval of COVID-19 vaccines for children, they have become a cause of anxiety and stress among some parents, who fear the accompanying risks and long-term side effects such as allergic reactions and infertility in the future. This misleading information can be a crucial obstacle in regard to vaccinating children who are exposed to unofficial news. This is the first study that evaluated parents’ perceptions of the use of COVID-19 vaccine for children in Saudi Arabia.

We found that 46.1% of the participants favored the COVID-19 vaccination, whereas 29.3% disagreed with providing vaccines to their children, and 24.4% were unsure. The present study results are close (but not similar) to the findings of studies performed in Turkey [21,22] but different from the conclusions of the USA, UK, China, and New Zealand studies [17,18,19,20]. Age, gender, occupation, income, and vaccination status have relatively high influences on parents’ views towards COVID-19 vaccination of their children.

Knowledge regarding COVID-19 and the vaccine was significantly correlated to gender (more towards males), education level, occupation, income, and vaccination status, as illustrated in Table 3. The most important reasons that directed parents toward the acceptance of the vaccine were the belief that the vaccine could protect against the disease and anxiety about their children being affected, which is consistent with the studies conducted in the USA and Turkey [17,21,22]. On the other hand, the most critical reason for parents’ refusal to vaccinate their children was the belief that children have natural immunity. Thus, they felt they did not need the vaccine, which is consistent with the USA study [17] and the UK study [18]. The second reason that prevented parents from vaccinating their children was a belief in the lack of reliability and validity of the vaccine. As shown in our study, 6% of parents refused to vaccinate their children because they believed that the goal of the vaccine was to profit, rather than to end the pandemic. This is somewhat similar to the UK study, which revealed that vaccine reluctance was aggravated by safety concerns due to accelerated development and cutting of corners [18]. In our study, 10% of participants thought the COVID-19 children’s vaccine was reliable, which is a very low percentage. This may be due to the influence of social media, unofficial news and rumors.

Goldman et al. [23] reported that a population-based vaccination program was started in Israel, which led to an increasing willingness among parents to vaccinate their children younger than 12 years against COVID-19. In Canada, the slow vaccination rate among the adult population was associated with a lower willingness to vaccinate children. Bagateli et al. [24] studied the prevalence and extent of COVID-19 vaccine hesitancy among parents of children and adolescents in Brazil. The authors reported that vaccine hesitancy was very low among caregivers, and many of the hesitant caregivers were willing to vaccinate their offspring against COVID-19.

### Strength and Weakness

This is the first study conducted in Saudi Arabia to evaluate parents’ perceptions in relation COVID-19 vaccination of children and the reasons behind their acceptance or refusal of the vaccine. One limitation of this study was that the sample size was not large enough to reflect the entire country’s population, and more than 80% of the participants were from Riyadh, thus not precisely reflecting the opinions of people from other cities.

## 5. Conclusions

There was a decrease in parents’ acceptance of the COVID-19 vaccination of children in Saudi Arabia. This requires more attention and focus from health providers to eliminate fear and anxiety among the parents through more educational programs and events to decrease resistance toward the vaccine. In addition, more studies are needed to ensure the vaccine’s safety. This study highlights the importance of offering the COVID-19 vaccination to children, including subjects who are uncertain about vaccines. More emphasis should be placed on the children’s vaccine campaign in order to increase the awareness of parents about the importance of the vaccine for children in Saudi Arabia.

## Figures and Tables

**Table 1:** Sociodemographic characteristics (n = 1304).

Variables		N	(%)
Age	18–29	70	(5.4)
	30–39	378	(29.0)
	40–49	542	(41.6)
	50 and more	314	(24.1)
Gender	Male	342	(26.2)
	Female	962	(73.8)
Education	Did not go to school	1	(0.1)
	Less than high school	26	(2.0)
	High school	130	(10.0)
	Diploma	16	(1.2)
	Bachelor’s	882	(67.6)
	Postgraduate	249	(19.1)
Occupation	Student	20	(1.5)
	Private sector	202	(15.5)
	Government sector	485	(37.2)
	Unemployed	418	(32.1)
	Retired	155	(11.9)
	Freelancer	24	(1.8)
Income	Less than 5000	63	(4.9)
	5000–14,999	500	(30.7)
	15,000–24,999	448	(34.4)
	25,000–50,000	285	(21.9)
	More than 50,000	108	(8.3)
Number of children between (5–12) years	1–2	965	(74.0)
	3–4	244	(18.7)
	More than four children	95	(7.3)
Vaccination status	non-immunized	32	(2.5)
	immunized with the first dose	84	(6.4)
	immunized with two doses	1121	(86.0)
	recovered immune	67	(5.1)
Have they been infected with the Coronavirus?	Myself	64	(4.9)
	No one from my family	746	(57.2)
	One of my family	281	(21.5)
	Me and one of my family	213	(16.3)

Data represented as number and percentage.

**Table 2:** Association of variables with the willingness of parents to give their children the vaccine (I intend to give my children who are 5–12 years old the COVID-19 vaccine).

Parameters	Agree	I Don’t Know	Disagree	Chi-Squared	*p*-Value
Parents age group					
18–29	28 (4.7%)	24 (7.5%)	18 (4.7%)	18.905	0.004 *
30–39	152 (25.2%)	104 (32.6%)	122 (31.9%)		
40–49	250 (41.5%)	127 (39.8%)	165 (43.1%)		
50 and more	172 (28.6%)	64 (20.1%)	78 (20.4%)		
Gender					
Male	198 (32.9%)	74 (23.2%)	70 (18.3%)	27.839	0.001 *
Female	404 (67.1%)	245 (76.8%)	313 (81.7%)		
Education					
Didn’t go to school	0 (0.0%)	0 (0.0%)	1 (0.3%)	12.551	0.250
Primary school	16 (2.7%)	5 (1.6%)	5 (1.3%)		
High school	73 (12.1%)	25 (7.8%)	32 (8.4%)		
Diploma	8 (1.3%)	5 (1.6%)	3 (0.8%)		
Bachelor’s	396 (65.8%)	218 (68.3%)	268 (70.0%)		
Postgraduate	109 (18.1%)	66 (20.7%)	74 (19.3%)		
Occupation					
Student	12 (2.0%)	4 (1.3%)	4 (1.0%)	25.747	0.004 *
Private sector	109 (18.1%)	44 (13.8%)	49 (12.8%)		
Government sector	212 (35.2%)	125 (39.2%)	148 (38.6%)		
Unemployed	168 (27.9%)	106 (33.2%)	144 (37.6%)		
Retired	91 (15.1%)	33 (10.3%)	31 (8.1%)		
Freelancer	10 (1.7%)	7 (2.2%)	7 (1.8%)		
Income (Saudi riyals per month)					
Less than 5000	27 (4.5%)	15 (4.7%)	21 (5.5%)	19.936	0.030 *
5000–14,999	180 (29.9%)	91 (28.5%)	129 (33.7%)		
15,000–24,999	197 (32.7%)	105 (32.9%)	146 (38.1%)		
25,000–50,000	147 (24.4%)	72 (22.6%)	66 (17.2%)		
More than 50,000	51 (8.5%)	36 (11.3%)	21 (5.5%)		
Number of children between (5–12) years					
1–2	439 (72.9%)	237 (74.3%)	289 (75.5%)	2.150	0.708
3–4	114 (18.9%)	58 (18.2%)	72 (18.8%)		
More than 4 children	49 (8.1%)	24 (7.5%)	22 (5.7%)		
vaccination status					
non-immunized	5 (0.8%)	3 (0.9%)	24 (6.3%)	96.268	0.001 *
immunized with first dose	16 (2.7%)	15 (4.7%)	53 (13.8%)		
immunized with two doses	559 (92.9%)	283 (88.7%)	279 (72.8%)		
recovered immune	22 (3.7%)	18 (5.6%)	27 (7.0%)		
Has been infected with the Coronavirus					
Me	32 (5.3%)	16 (5.0%)	16 (4.2%)	1.962	0.923
No one from my family	349 (58.0%)	184 (57.7%)	213 (55.6%)		
One of my family	123 (20.4%)	68 (21.3%)	90 (23.5%)		
Me and one of my family	98 (16.3%)	51 (16.0%)	64 (16.7%)		

Data were represented as number and percentage, Chi-squared test: * Significant at *p* ≤ 0.05.

**Table 3:** Correlation of variables to assess the knowledge regarding COVID-19 and willingness to vaccinate children for COVID-19.

Variables		Mean	S. D	F Test	*p*-Value
Age	18–29	78.4	0.207	2.099	0.099
	30–39	76.9	0.213		
	40–49	76.2	0.211		
	50 and more	75.8	0.226		
Gender	Male	79.5	0.218	13.450	0.001*
	Female	74.5	0.213		
Occupation	Student	78.8	0.244	3.705	0.002 *
	Private sector	78.6	0.193		
	Government sector	76.9	0.219		
	Unemployed	74.5	0.210		
	Retired	74.4	0.226		
	Freelancer	61.1	0.238		
Income	Less than 5000	67.9	0.280	6.696	0.001 *
	5000–14,999	74.0	0.221		
	15,000–24,999	74.8	0.207		
	25,000–50,000	80.0	0.198		
	More than 50,000	79.9	0.205		
Number of children 5–12 years age	1–2	76.2	0.209	0.998	0.369
	3–4	75.2	0.226		
	More than four children	73.2	0.249		
Vaccination status	non-immunized	53.4	0.274	24.65	0.001 *
	immunized with the first dose	66.6	0.255		
	immunized with two doses	77.7	0.203		
	recovered and immune	66.8	0.223		
Have they been infected with the Coronavirus?	Myself	72.2	0.225	1.176	0.317
	No one from my family	76.6	0.212		
	One of my family	75.6	0.218		
	Me and one of my family	74.5	0.217		

Data represented as mean and standard deviation (SD); * Significant at *p* ≤ 0.05.

## Data Availability

May be provided on reasonable request to the corresponding author.
